# A bibliometric analysis of chronic subdural hematoma since the twenty-first century

**DOI:** 10.1186/s40001-022-00959-7

**Published:** 2022-12-27

**Authors:** Rundong Chen, Yanpeng Wei, Xiaolong Xu, Renkun Zhang, Yuhao Tan, Guanghao Zhang, Hongwei Yin, Dongwei Dai, Qiang Li, Rui Zhao, Qinghai Huang, Yi Xu, Pengfei Yang, Jianmin Liu, Qiao Zuo

**Affiliations:** Neurovascular Center, Changhai Hospital, Naval Medical University, #168 Changhai Road, Shanghai, 200433 China

**Keywords:** Chronic subdural hematoma, Middle meningeal artery embolization, Surgical treatment, Medication, Bibliometric analysis, CiteSpace

## Abstract

**Background:**

Chronic subdural hematoma (CSDH) is a common disease that forms between the dura and arachnoid membranes of the brain. With the development of medications and surgery, significant progress has been made in the diagnosis and treatment of CSDH. However, there is no comprehensive analysis available on CSDH-related studies published in the literature. This study aimed to collect and analyze CSDH-related studies published since the twenty-first century using bibliometric analysis and to summarize the current status of research in this field for the sake of providing systematic data for further study of CSDH.

**Methods:**

CSDH-related studies were searched in the Web of Science Core Collection (WoSCC) database using the Medical Subject Heading (MeSH) term ‘chronic subdural hematoma’. Data analysis and visualization were performed by R and CiteSpace software.

**Results:**

This study retrieved 1424 CSDH-related articles published since the beginning of the twenty-first century. There was a general increase in both the number of published articles and the mean number of citations. The authors, institutions and journals that contributed the most to the field of CSDH were Jianning Zhang, Tianjin Medical University, and world neurosurgery, respectively. The reference co-citation network identified 13 clusters with significant modularity Q scores and silhouette scores (*Q* = 0.7124, *S* = 0.8536). The major research categories were (1) evolution of the therapeutic method and (2) the etiology and pathology of CSDH. Keyword analysis revealed that ‘middle meningeal artery embolization’ was the latest burst keyword.

**Conclusions:**

This study identified the most influential countries, authors, institutions and journals contributing to CSDH research and discussed the hotspots and the latest subjects of CSDH research.

**Supplementary Information:**

The online version contains supplementary material available at 10.1186/s40001-022-00959-7.

## Introduction

Chronic subdural hematoma (CSDH) is a common disease occurring between the dura and arachnoid membrane [1]. The incidence of CSDH ranges from 1.7 to 20.6 per 100000 persons/year [2, 3]. The risk of CSDH seems to be increasing gradually, partly due to the aging population and the increased use of antiplatelet and anticoagulant agents [4]. CSDH is more common in elderly patients who are more susceptible to cerebral atrophy [5]. This increases the incidence of CSDH to 58 per 100,000 persons/year in the age group over 65 years [6]. For small CSDH patients who are asymptomatic or have mild symptoms, drug therapy is always the preliminary consideration. Several studies [4, 7] demonstrated that statin medications could reduce the size of CSDHs. Another study [8] reported that glucocorticoids could also reduce the recurrence of CSDH, but the functional outcome in the glucocorticoid group was no better than that in the placebo group. For patients with large and symptomatic CSDHs or progressive neurological deterioration caused by CSDHs, hematoma evacuation is required, and the outcomes are generally favorable [8]. However, the high recurrence rate of CSDH after surgery is still a clinical challenge, accounting for 10–20% of reported cases [3]. Endovascular middle meningeal artery (MMA) embolization is a new minimally invasive option for CSDH to reduce the possibility of hematoma recurrence [9]. However, the effectiveness and safety of different treatment strategies for CSDH still need to be further explored.

Since the twenty-first century, significant progress has been made in the field of CSDH, leading to an increase in the number of publications in this field. Bibliometrics uses statistical methods to analyze publications, especially those in the scientific lines. Bibliometric mapping enables data to be presented in a more comprehensible manner, thus providing researchers with relatively macrolevel information.

This study aimed to qualitatively discuss publications on CSDH since the twenty-first century by summarizing the major research subjects and frontiers using the bibliometric analysis platform CiteSpace, hoping that the results obtained could provide some unique insights into the development of CSDH over the last two decades.

## Materials and methods

### Data collection

We searched publications from the Web of Science Core Collection (WoSCC) through the Science Citation Index Expanded (SCI-E). We mainly searched the Medical Subject Heading (MeSH) term “chronic subdural hematoma” on August 8, 2022. The language was limited to ‘English’. The document types were ‘articles’ and ‘review’. The time span was after 2000. Complete associated data, including titles, authors, institutions, countries, journals, abstracts, keywords, journals, references, and citations, were exported in TXT format for analysis.

### Data analysis

The search result records were analyzed by R software (4.1.3) and CiteSpace software (6.1. R2), which were used to visually represent the scientometric analysis results, facilitating visual interpretations. Bibliographic coupling was referred to as the situation, where two references were cited together. Therefore, the coupling strength between publications indicated that these two publications were more related and had more similar research subjects. The citation network formed by bibliographic coupling was a static structure, which could be visualized and clustered to obtain a deeper interpretation. The ‘bibliometrix’ R package in R software was used to summarize the primary information, country scientific production, and the cumulative occurrence of journal articles in this study. CiteSpace was used to explore networks of co-cited references and co-occurring keywords, as well as collaboration networks between countries, authors, institutions, and journals. Burst detection could obtain nodes that burst over a period of time, which represents a topic that researchers were interested in during this period. The timeline map placed the publications in the same cluster on the same horizontal line, through which we were able to obtain the number of publications in each cluster and the time width of the research. The number of publications in the cluster represents the importance of the cluster in this field, and the length of the time span represents the time when the cluster attracted interest.

CiteSpace provides a variety of important metrics, such as Freeman's betweenness centrality metric, which can identify key hubs [10]. The burstness of the frequency can detect the occurrence and duration of abrupt changes in frequency. The modularity (Q score) of a network can measure how well the network is divided into modules or clusters, while the silhouette (S score) is a way to explain and verify the consistency of data within clusters [11]. When the Q score is greater than 0.3, the cluster structure is considered to be statistically significant, with higher values indicating a more robust cluster structure. A silhouette coefficient of 0.3 or higher indicates a homogeneous network, while a silhouette coefficient of 0.5 or higher indicates a plausible network. A silhouette coefficient of 0.7 or higher indicates a highly plausible network. A silhouette coefficient close to 1 indicates that the corresponding cluster is relatively isolated.

## Results

### General overview

**Fig. 1:**
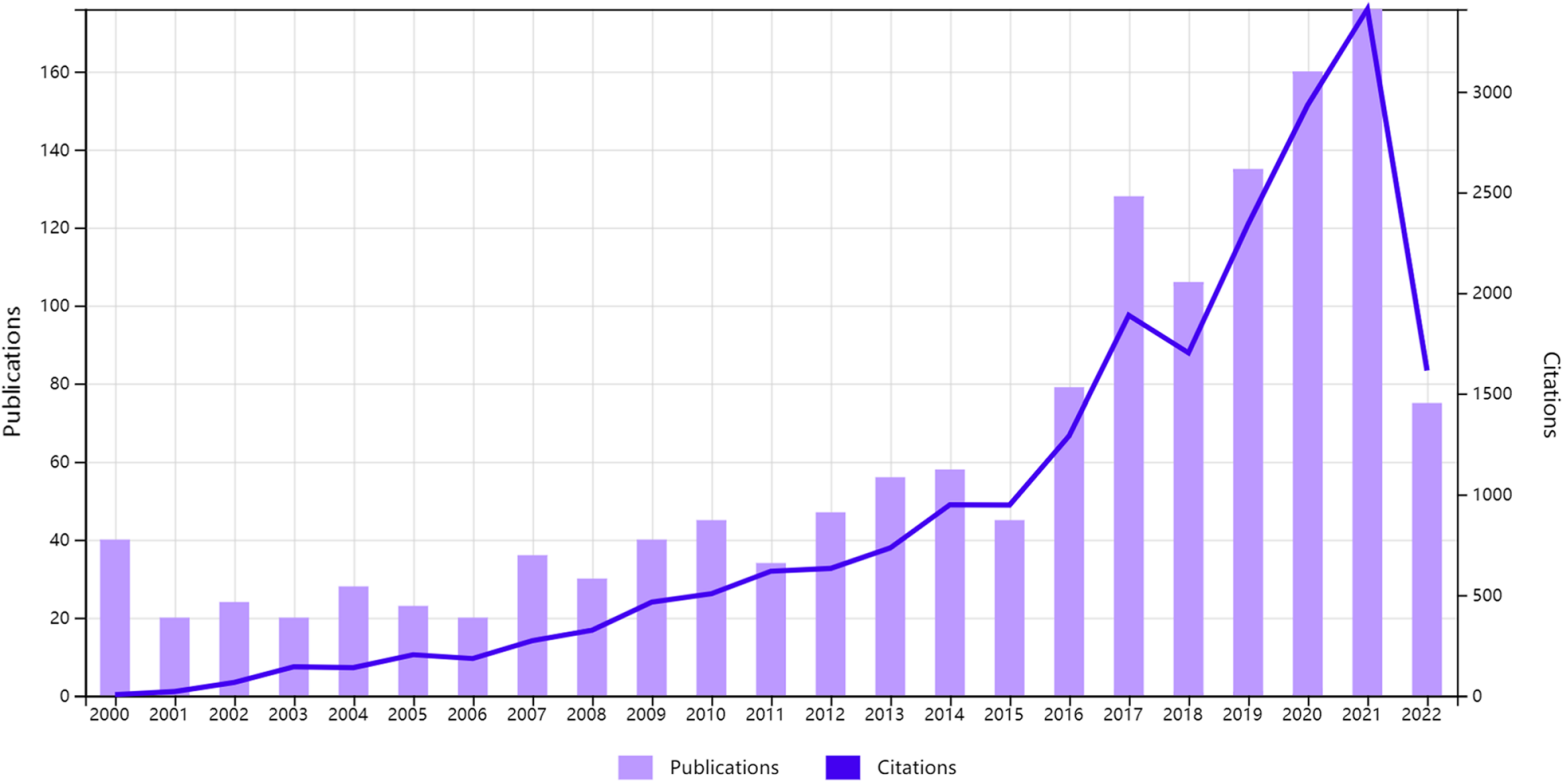
Annual scientific production and citations

Since the beginning of the twenty-first century, 1424 publications on CSDH have been included in this study, including 1252 original articles and 172 review articles (Fig. 1). The number of published articles and the mean number of citations showed a generally increasing trend.

### Co-cited reference analysis

**Fig. 2:**
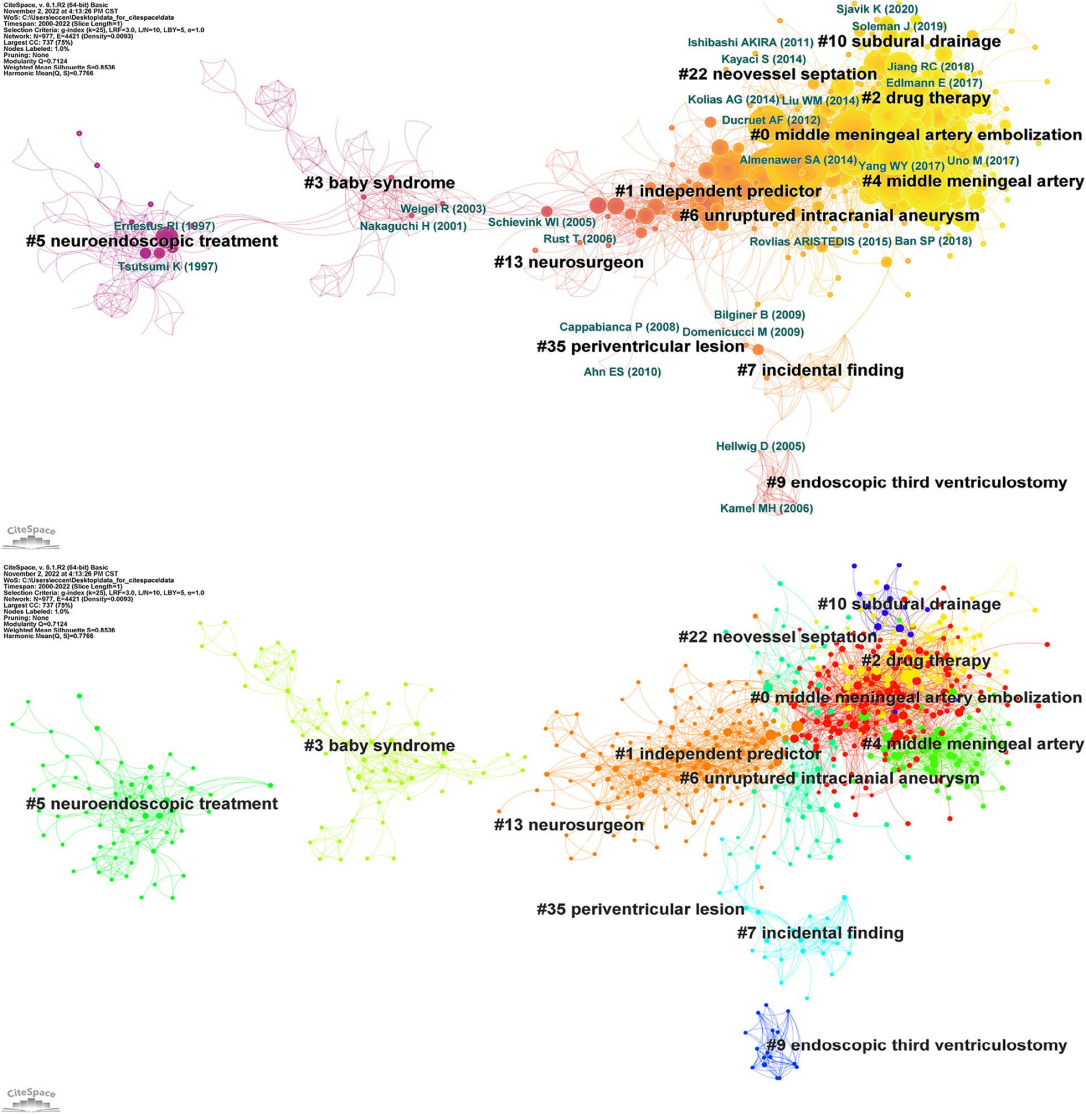
**A** Co-citation reference network with cluster visualization. **B** Visualization map of the corresponding clusters. Publication topics of the same type are clustered in the same color block

The maps of reference co-citations and corresponding clusters were constructed by CiteSpace (Fig. 2A, B). Thirteen different clusters were identified in this network with significant modularity Q scores and silhouette scores (*Q* = 0.7124, *S* = 0.8536). The largest cluster (#0) had 182 members and a silhouette value of 0.681. It was labeled ‘middle meningeal artery embolization’. Ryota Tamura was the author of the most relevant citation to the cluster [12]. The 2nd largest cluster (#1) had 146 members and a silhouette value of 0.865. It was labeled ‘independent predictor’. David Balser was the author of the most relevant citation to the cluster [13]. The 3rd largest cluster (#2) had 83 members and a silhouette value of 0.852. It was labeled ‘drug therapy’. Joshua S Catapano was the author of the most relevant citation to the cluster [14].

### Keyword analysis

**Fig. 3:**
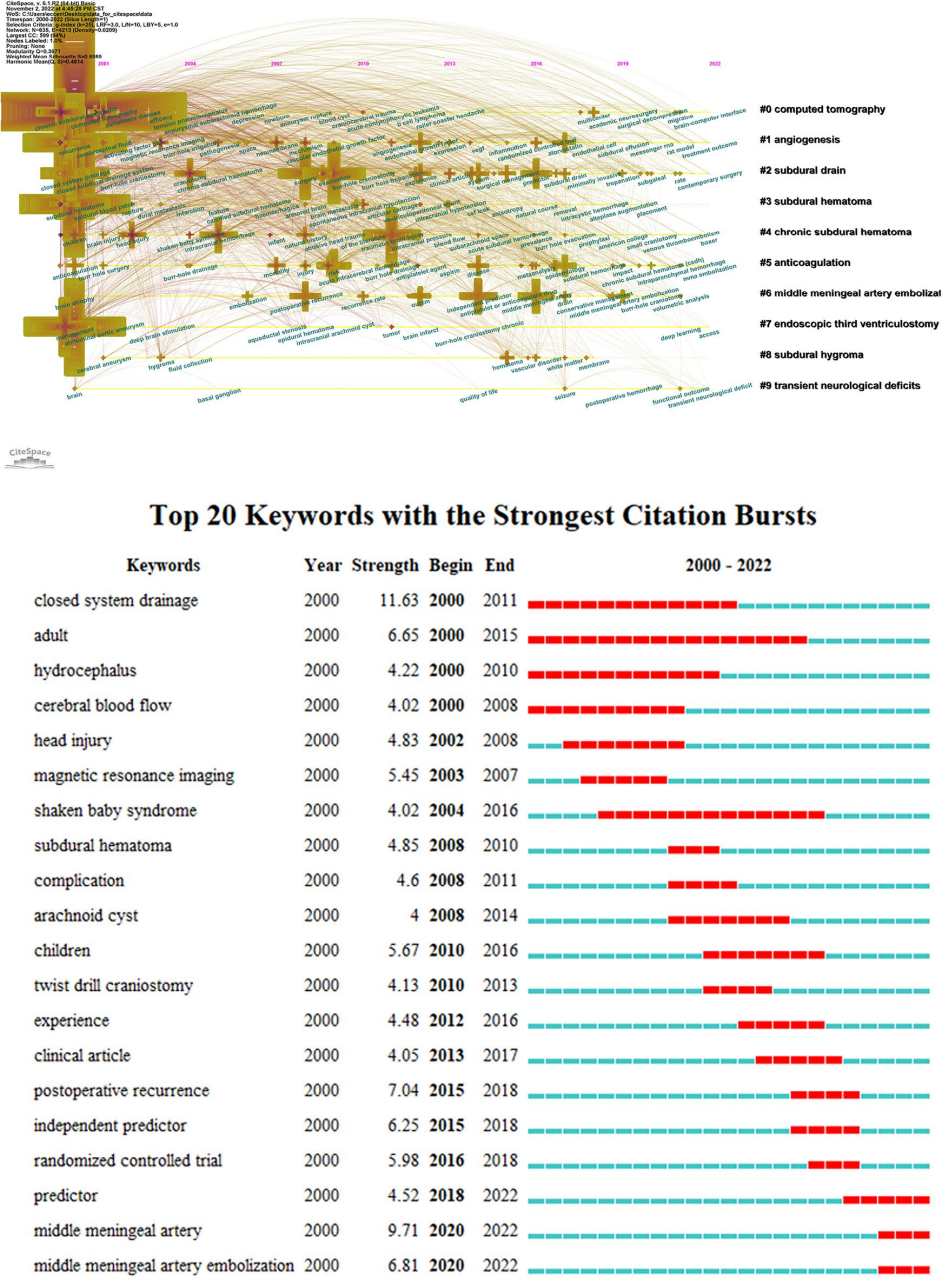
**A** Timeline visualization of co-occurring author keyword networks since the beginning of the twenty-first century. The size of a cross is proportional to burst keyword co-occurrence. The clusters are labeled in black to the right of the timeline maps. **B** Top 20 keywords with the strongest citation bursts

The timeline of the co-occurring keyword network was extracted by CiteSpace (Fig. 3A). Ten clusters of co-occurring keywords were identified with a modularity Q score = 0.3671 and silhouette score = 0.6989. The most crucial cluster was ‘clopidogrel’, followed by ‘computered tomography’, ‘angiogenesis’, ‘subdural drain’, ‘subdural hematoma’, ‘chronic subdural hematoma’, ‘anticoagulation’, ‘middle meningeal artery embolization’, ‘endoscopic third ventriculostomy’, ‘subdural hygroma’ and ‘transient neurological deficits’. Moreover, keyword bursts represented keywords that were frequently cited over a period of time (Fig. 3B). The earliest burst keywords were ‘closed system drainage’, ‘adult’, ‘hydrocephalus’, and ‘cerebral blood flow’, which began in 2000 and lasted for 15 years. Subsequently, emerging keywords were ‘head injury’, ‘magnetic resonance imaging’, and ‘shaken baby syndrome’. These keywords further evolved into ‘subdural hematoma’, ‘complication’, ‘arachnoid cyst’, ‘children’, and ‘twist drill craniostomy’, and then ‘experience’, ‘clinical article’, ‘postoperative recurrence’, and ‘randomized controlled trial’. More recently, these keywords have become ‘predictor’, ‘middle meningeal artery’, and ‘middle meningeal artery embolization’.

### Country analysis

**Fig. 4:**
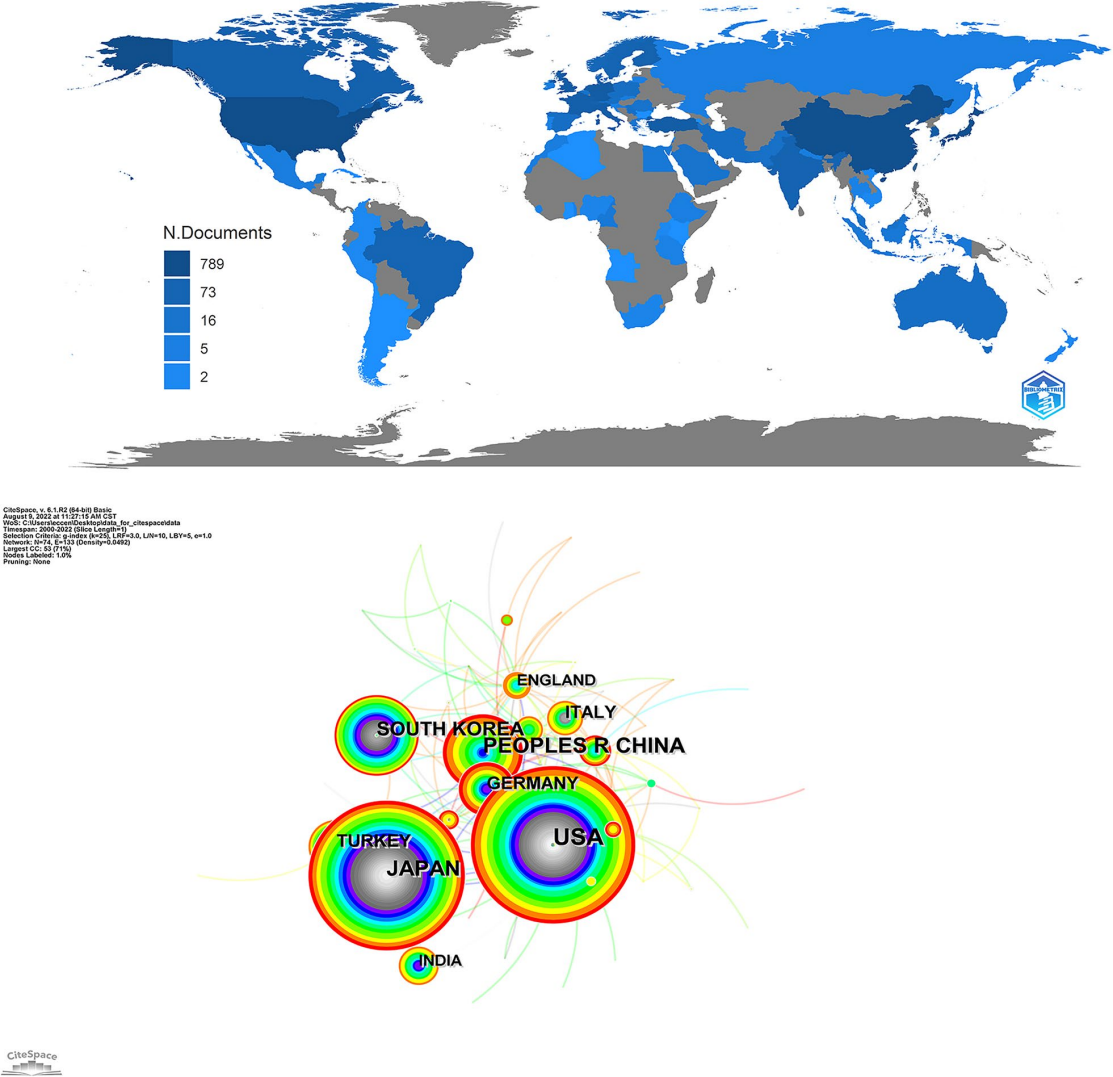
**A** Country-specific production. Dark blue = high productivity. Gray = no documents. **B** Country cooperation network map

Based on the analysis of cooperation networks, 74 countries or regions were identified. The United States contributed the most publications (*n* = 319), followed by Japan (*n* = 236), China (*n* = 172), South Korea (*n* = 95), and Germany (*n* = 76) (Additional file 1: Table S1). The country scientific production map is shown in Fig. 4A, and the cooperation networks across countries are mapped in Fig. 4B.

### Author and institution analysis

**Fig. 5:**
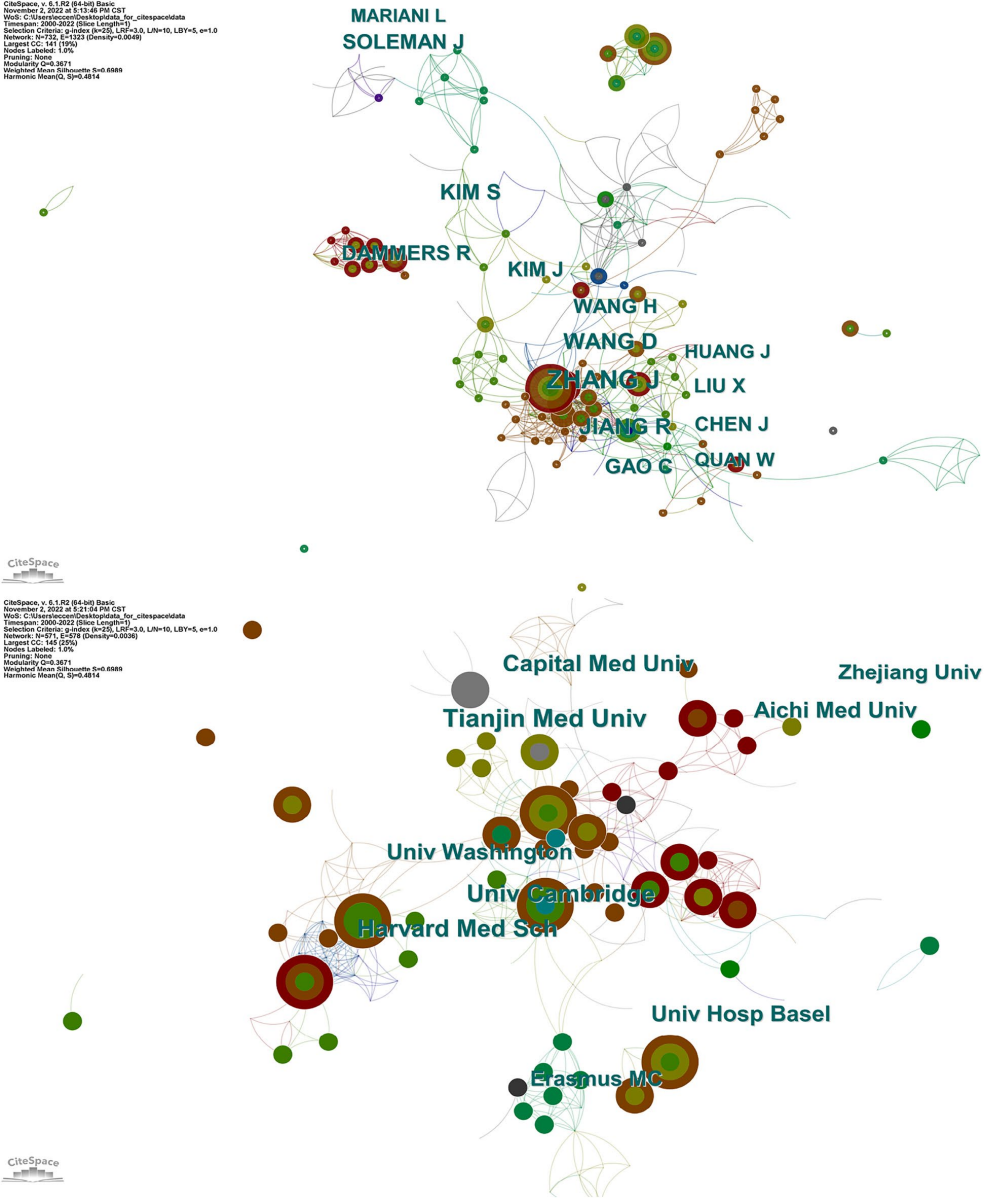
**A** Author cooperation network map. **B** Institution cooperation network map

The cooperation network between authors is shown in Fig. 5A, and the top 10 influential authors are shown in Additional file 2: Table S2. The results showed that Jianning Zhang had 33 publications, ranking first, followed by Dong Wang (*n* = 20), Rongcai Jiang 11 (*n* = 18), Soo-Han Kim (*n* = 16), and R Dammers (*n* = 14). The cooperation network between institutions is shown in Fig. 5B. The top 5 institutions with citation counts are Tianjin Medical University (*n* = 20), the University of Cambridge (*n* = 17), Harvard Medical School (*n* = 16), the Capital Medical University (*n* = 15), and the University Hospital Basel (*n* = 13) (Additional file 3: Table S3).

### Journal analysis

**Fig. 6:**
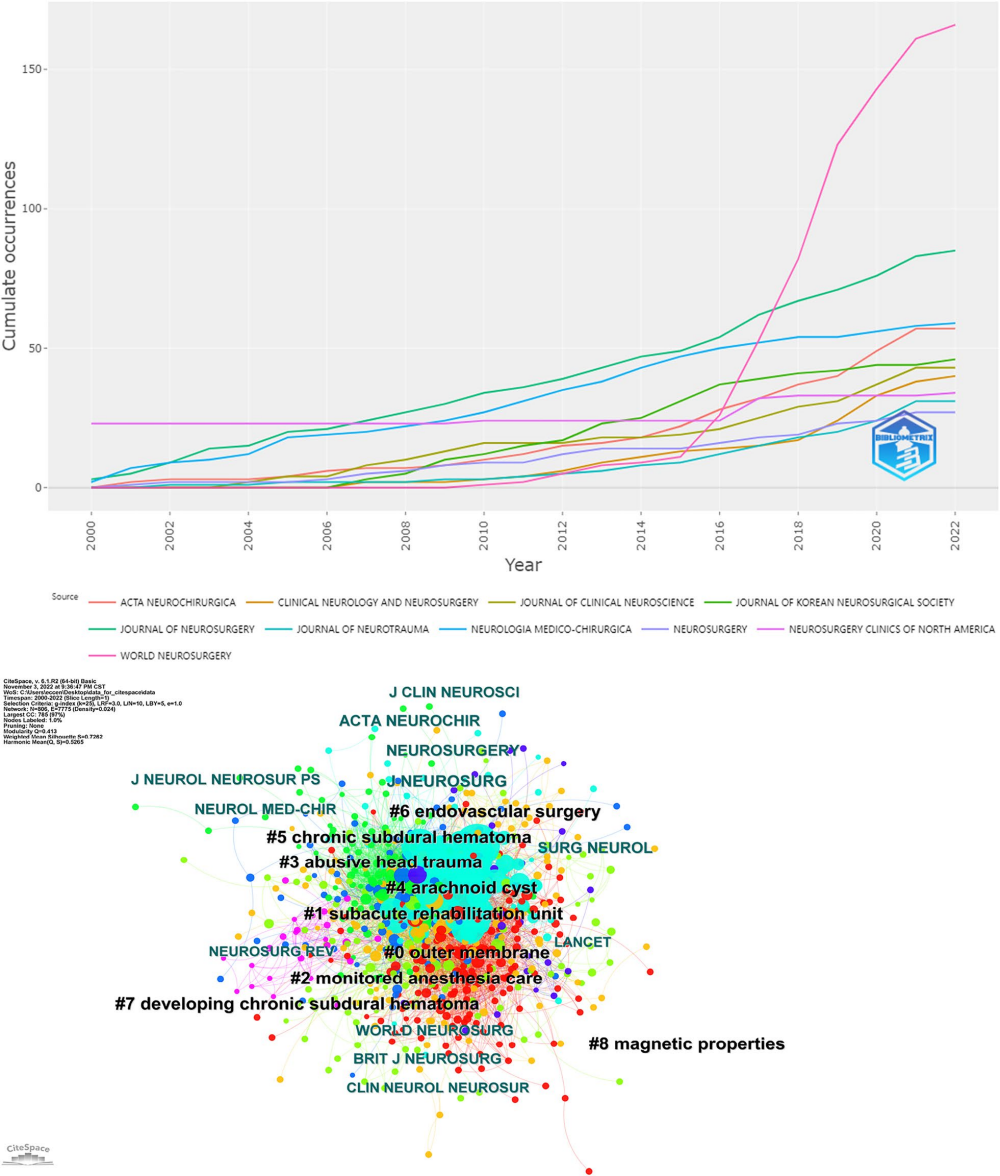
**A** Source growth of publications. **B** Co-cited journal network with cluster visualization

The top five journals with the most references are the World Neurosurgery (*n* = 166), Journal of Neurosurgery (*n* = 85), Neurologia medico-chirurgica (*n* = 59), Acta Neurochirurgica (*n* = 57), and Journal of Korean Neurosurgical Society (*n* = 46) (Fig. 6A). The co-cited journal network is shown in Fig. 6B. The Journal of Neurosurgery, Neurosurgery, Acta Neurochirurgica, Surgical Neurology, and World Neurosurgery are the top five journals with the highest number of citations (Additional file 4: Table S4).

## Discussion

This study first revealed the development tendency of CSDH by bibliometric analysis. The number of publications showed a generally increasing trend, involving multiple subject areas, including neurosurgery, cerebrovascular, neurointervention, pathophysiology, and management. The top 10 most locally cited publications representing the most prominent are shown in Table 1, which provides a new perspective to explore the impact of these papers on CSDH [4, 5, 15–22]. These publications were mainly concerned with the pathophysiology, epidemiology, diagnosis, and management of CSDH. Among them, the choice of treatment scheme was the topic winning the most discussion. At first, surgical treatment was widely discussed, and in the past 5 years, more attention has been paid to middle meningeal artery embolization and drug treatment.

**Table 1:** Top 10 most locally cited publications

Rank	Frequent	Burst	Degree	Centrality	Sigma	Author	Years	Source	Title	Cluster
1	133	19.25	27	0.02	1.33	Ellie Edlmann	2017	Journal of neuroinflammation	Pathophysiology of chronic subdural haematoma: inflammation, angiogenesis and implications for pharmacotherapy	2
2	77	24.71	39	0.08	7.12	Saleh A Almenawer	2014	Annals of surgery	Chronic subdural hematoma management: a systematic review and meta-analysis of 34,829 patients	1
3	72	10.55	24	0.04	1.47	Wuyang Yang	2017	Neurosurgery clinics of North America	Chronic subdural hematoma: epidemiology and natural history	0
4	71	11	38	0.01	1.1	Seung Pil Ban	2018	Radiology	Middle meningeal artery embolization for chronic subdural hematoma	4
5	65	31.49	38	0.1	17.51	Andrew F Ducruet	2012	Neurosurgical review	The surgical management of chronic subdural hematoma	1
6	64	23.6	14	0.01	1.26	Weiming Liu	2014	Journal of neurosurgery	Chronic subdural hematoma: a systematic review and meta-analysis of surgical procedures	4
7	58	3.95	28	0.05	1.19	Henrique Seiji Ivamoto	2016	World neurosurgery	Surgical treatments for chronic subdural hematomas: a comprehensive systematic review	4
8	56		42	0.02	1	Thomas W Link	2019	Neurosurgery	Middle meningeal artery embolization for chronic subdural hematoma: a series of 60 cases	4
9	55	8.31	29	0.01	1.09	Rongcai Jiang	2018	JAMA neurology	Safety and efficacy of atorvastatin for chronic subdural hematoma in chinese patients: a randomized clinical trial	2
10	54	19.87	10	0.01	1.12	Angelos G Kolias	2014	Nature reviews neurology	Chronic subdural haematoma: modern management and emerging therapies	6

References co-cited with the corresponding clustering networks described the associations between 13 different clusters on CSDH. Two major categories of research subjects were identified. The most important category was the evolution of treatment methods (Cluster #5 (neuroendoscopic treatment), Cluster #13 (neurosurgeon), and now mainly focused on Cluster #10 (subdural drainage), Cluster #0 (middle meningeal artery embolization), Cluster #2 (drug therapy)). The second major category was concerned with etiology and pathology (Cluster #3 (baby syndrome), Cluster #1 (independent predictor), Cluster #9 (endoscopic third ventriculostomy), Cluster #7 (incidental finding), Cluster #22 (neovessel septation), Cluster #35 (periventricular lesion), and Cluster #6 (unruptured intracranial aneurysm). The burst keywords that could identify the latest trends of research determined the same results. The first and strongest burst hotspot was ‘closed system drainage’, which began in 2000 and lasted for 11 years. Subsequently, there were extensive discussions on magnetic resonance imaging (MRI), complications, postoperative recurrence, and randomized controlled trials. More recently, the hotspot has been turned into ‘middle meningeal artery embolization’, which took second place in burst strength.

These two trends play a role in explaining the long-term development of CSDHs. Neurosurgeons initiated surgical treatment of CSDH patients as early as the last century using burr holes, subdural peritoneal shunt, craniotomy, subdural tap, stripping of membranes, or their combination [23]. However, what surgical modalities can provide the optimal outcome remains controversial [24]. To the best of our knowledge, BHC offers the lowest recurrence rate with a manageable complication rate and is, therefore, considered the treatment of first choice for CSDH [25]. MMA embolization, as a less invasive method, has received more attention [9, 14, 26]. Since the membrane of CSDHs is derived from the dura mater, the development of new ‘leaky’ blood vessels from the membrane is currently considered a cause contributing to CSDH evolution [15]. Concerning the etiology and pathology of CSDH, the early theory was that traumatic injury led to tearing of the bridging veins, causing venous blood accumulation in the subdural space [27]. However, this theory has long been disputed. Another theory hypothesizes that inflammation is the key factor [15]. It has long been accepted that a persistent inflammatory response after injury results in the proliferation of dural border cells, the formation of two new membranes, and the development of new ‘leaky’ blood vessels, thus allowing blood exudation and fluid accumulation into the subdural cavity [22, 28]. With the revelation of this theory, atorvastatin has been shown to have a range of properties against CSDH, especially in reducing inflammation-induced vascular leakage and promoting angiogenesis.

According to country analysis, the United States had the highest number of publications, with a centrality of 0.52, which implied that the USA dominated the field of CSDH research. It was likely that the high number of research institutions and the significant investment in research were contributing factors to the high ranking of the country in question. Cooperative networks, including co-author networks of countries and institutions, were analyzed. Researchers could visualize the impact of research teams on scientific knowledge and evaluate potential research collaborators. The author, institution, and journal that contributed the most to the field of CSDH were Jianning Zhang, Tianjin Medical University, and World Neurosurgery, respectively. Jianning Zhang and Tianjin Medical University made great contributions to atorvastatin as a nonsurgical alternative treatment for CSDH patients [4, 29, 30]. A randomized clinical trial published by Zhang Jianning recruiting 254 patients with CSDH reported that neurological function was significantly improved in 45.9% of the patients who used atorvastatin versus 28.6% in the placebo group. In addition, 11.2% of patients who used atorvastatin and 23.5% of patients who used placebo underwent surgical treatment during the trial for an enlarging hematoma and/or a deteriorating clinical condition [4], and the results suggested that atorvastatin, as a nonsurgical treatment, may be a safe and effective nonsurgical alternative for the treatment of CSDH patients. World Neurosurgery, as the journal with the most articles in the field of CSDH, suggests that it is possible to obtain cutting edge information in this journal and provide researchers with an appropriate journal to submit their articles [3, 9, 31].

Although the understanding of CSDHs has gradually deepened, there are still many directions that deserve attention. MMA embolization was detected as a burst keyword in 2020 and became an increasingly popular treatment option for CSDH patients who are unable to tolerate surgical treatment due to anticoagulant and/or antiplatelet medication or poor physical condition [21, 26]. It was also shown to be effective in reducing the possibility of recurrence. This hotspot could potentially last for years. In addition, a lack of understanding of the underlying pathogenesis in the CSDH field has hampered the development of more effective treatments; further study of its etiology and pathology is needed. This study could assist scholars from various parts of the world in determining the leading experts and publications in the CSDH field and encourage wider cooperation.

There were also some limitations of this study. We only retrieved publications from the WoSCC database considering the comprehensiveness of the information in the publication, which may lead to incomplete publication collection.

## Conclusions

This bibliometric study first analyzed CSDH-related publications objectively. The results showed that the number of publications has increased since 2000. We identified the most influential countries, authors, institutions, and journals, as well as hotspots and the latest research subjects, mainly for middle meningeal artery embolization. More collaboration is needed between institutions. This study could also guide clinicians and scholars engaged in the study of CSDH in the future.

## Supplementary Information

**Additional file 1: Table S1.** Top 10 countries with most publications.

**Additional file 2: Table S2.** Top 10 authors with most publications.

**Additional file 3: Table S3.** Top 10 institutions with most publications.

**Additional file 4: Table S4.** Top 10 journals with most publications.

## Data Availability

All data and materials can be accessed in WoSCC.
