# Supplementary material for: A bibliometric analysis of chronic subdural hematoma since the twenty-first century
Source: Eur J Med Res. 2022 Dec 27;27:309. doi: 10.1186/s40001-022-00959-7 (PMC9793598; doi:10.1186/s40001-022-00959-7)
Supplement: Supplementary file 1 — Additional file 1: Table S1. Top 10 countries with most publications. [file 40001_2022_959_MOESM1_ESM.docx]

**Table S1** Top 10 countries with most publications

| Rank | Counts | Centrality | Country |
| --- | --- | --- | --- |
| 1 | 319 | 0.52 | United States of America |
| 2 | 236 | 0 | Japan |
| 3 | 172 | 0.08 | People's Republic of China |
| 4 | 95 | 0 | South Korea |
| 5 | 76 | 0.11 | Germany |
| 6 | 67 | 0.04 | Turkey |
| 7 | 55 | 0 | Italy |
| 8 | 50 | 0 | India |
| 9 | 48 | 0.29 | England |
| 10 | 44 | 0.09 | Canada |
