# Supplementary material for: A bibliometric analysis of chronic subdural hematoma since the twenty-first century
Source: Eur J Med Res. 2022 Dec 27;27:309. doi: 10.1186/s40001-022-00959-7 (PMC9793598; doi:10.1186/s40001-022-00959-7)
Supplement: Supplementary file 2 — Additional file 2: Table S2. Top 10 authors with most publications. [file 40001_2022_959_MOESM2_ESM.docx]

**Table S2** Top 10 authors with most publications

| rank | counts | centrality | Author |
| --- | --- | --- | --- |
| 1 | 33 | 0.03 | Jianning Zhang |
| 2 | 20 | 0.01 | Dong Wang |
| 3 | 18 | 0.01 | Rongcai Jiang |
| 4 | 16 | 0.01 | Seon-Hwan Kim |
| 5 | 14 | 0 | Jehuda Soleman |
| 6 | 14 | 0 | Chuang Gao |
| 7 | 14 | 0 | Ruben Dammers |
| 8 | 14 | 0.02 | Jong-Soo Kim |
| 9 | 13 | 0 | Xuanhui Liu |
| 10 | 13 | 0 | Haifeng Wang |
