# Supplementary material for: A bibliometric analysis of chronic subdural hematoma since the twenty-first century
Source: Eur J Med Res. 2022 Dec 27;27:309. doi: 10.1186/s40001-022-00959-7 (PMC9793598; doi:10.1186/s40001-022-00959-7)
Supplement: Supplementary file 3 — Additional file 3: Table S3. Top 10 institutions with most publications. [file 40001_2022_959_MOESM3_ESM.docx]

**Table S3** Top 10 institutions with most publications

| Rank | Counts | Centrality | Institution |
| --- | --- | --- | --- |
| 1 | 20 | 0.04 | Tianjin Medical University |
| 2 | 17 | 0.04 | University of Cambridge |
| 3 | 16 | 0.07 | Harvard Medical School |
| 4 | 15 | 0.01 | Capital Medical University |
| 5 | 13 | 0 | University Hospital Basel |
| 6 | 13 | 0.05 | University of Washington |
| 7 | 13 | 0 | Aichi Medical University |
| 8 | 11 | 0 | Erasmus University Medical Center |
| 9 | 11 | 0 | Zhejiang University |
| 10 | 10 | 0.05 | Leiden University |
