# Supplementary material for: A bibliometric analysis of chronic subdural hematoma since the twenty-first century
Source: Eur J Med Res. 2022 Dec 27;27:309. doi: 10.1186/s40001-022-00959-7 (PMC9793598; doi:10.1186/s40001-022-00959-7)
Supplement: Supplementary file 4 — Additional file 4: Table S4. Top 10 journals with most publications. [file 40001_2022_959_MOESM4_ESM.docx]

**Table S4** Top 10 journals with most publications

| Rank | Counts | Centrality | Journal |
| --- | --- | --- | --- |
| 1 | 1175 | 0 | Journal of Neurosurgery |
| 2 | 875 | 0 | Neurosurgery |
| 3 | 818 | 0 | Acta Neurochirurgica |
| 4 | 689 | 0 | Surgical Neurology |
| 5 | 574 | 0.01 | World Neurosurgery |
| 6 | 543 | 0 | Neurologia medico-chirurgica |
| 7 | 535 | 0 | Journal of Clinical Neuroscience |
| 8 | 533 | 0.01 | Journal of Neurology, Neurosurgery and Psychiatry |
| 9 | 531 | 0.01 | British Journal of Neurosurgery |
| 10 | 459 | 0.01 | Neurosurgical Review |
